# *OsLAP3/OsSTRL2*, encoding a rice strictosidine synthase, is required for anther cuticle formation and pollen exine patterning in rice

**DOI:** 10.3389/fpls.2024.1508828

**Published:** 2025-01-20

**Authors:** Dai-bo Chen, Ran Zhou, Hui-min Wang, Pei-pei Zhang, Zheng-fu Yang, Dan-dan Xuan, Ying-xin Zhang, Xiao-deng Zhan, Li-yong Cao, Shi-hua Cheng, Lian-ping Sun

**Affiliations:** ^1^ State Key Laboratory of Rice Biology and Breeding, National Center of Rice Improvement, China National Rice Research Institute, Hangzhou, China; ^2^ National Nanfan Research Institute (Sanya), Chinese Academy of Agricultural Sciences, Sanya, China; ^3^ Jiangxi Academy of Agricultural Sciences, Nanchang, China; ^4^ State Key Laboratory of Subtropical Silviculture, Zhejiang A&F University, Hangzhou, Zhejiang, China

**Keywords:** rice, male sterility, *OsLAP3*, pollen exine, anther development

## Abstract

The formation of the anther wall and the development of pollen processes, central to rice fertility and yield, are highly dependent on the synthesis and accumulation of lipid polymers. Although several regulatory factors related to lipid biosynthesis during pollen wall development have been identified, the molecular mechanisms controlling these processes remain poorly understood. In this study, a male-sterile rice mutant, *lap3*, was identified, characterized by normal vegetative growth but complete male sterility due to delayed programmed cell death (PCD) in tapetal cells and defects in anther cuticle and pollen exine formation. Map-based cloning revealed that *OsLAP3* is a new allele of the strictosidine synthase-like gene, *OsSTRL2*. Functional analysis, including complementation and CRISPR/Cas9-based gene editing, confirmed that the 2-nucleotide deletion in the *OsLAP3* is responsible for the male sterility phenotype. *OsLAP3* is homologous to the maize *ZmMS45*, the core recessive nuclear sterile gene of maize Seed Production Technology (SPT), and localizes to the endoplasmic reticulum and plays a conserved role in anther development and pollenformation. Gene expression analysis revealed a significant downregulation of key genes involved in anther development and sporopollenin biosynthesis in *lap3* anthers. Furthermore, lipid profiling demonstrated a marked reduction in both wax and cutin content. These findings establish *OsLAP3* as a critical regulator of fatty acid synthesis and highlight its role in anther cuticle formation and pollen exine development. The findings of this study provide valuable insights into the molecular regulation of lipid biosynthesis during rice male reproductive development and offer potential applications for hybrid rice breeding.

## 1 Introduction

Male sterility in rice is a critical aspect of reproductive development and forms the foundation for hybrid rice breeding, which significantly enhances crop yield (Yuan, 2014). Multiple factors contribute to male sterility, involving key processes such as microspore mother cell development, meiosis, tapetum development and degradation, nutrient synthesis and transport within the anther wall, pollen wall formation, starch accumulation, and anther dehiscence (Fu et al., 2014; Shi et al., 2011; Mu et al., 2009; Ranjan et al., 2017). Among these processes, the programmed cell death (PCD) and synthesis and transport of nutrients in the anther wall have been the most extensively studied (Zhang and Yang, 2014). These events are crucial for the proper formation and maturation of pollen cells during the mid-to-late stages of anther development and directly impact male reproductive success in rice.

Anther development in rice is governed by a complex network of transcription factors (TFs) that regulate PCD in the tapetum, a necessary process for pollen development and fertility. Several TFs, including Tapetum Degeneration Retardation (TDR) (Li et al., 2006), Eternal Tapetum 1 (EAT1) (Niu et al., 2013), and TDR Interacting Protein 2 (TIP2) (Fu et al., 2014), play crucial roles in regulating tapetal PCD and pollen wall formation (Abbas et al., 2021). TDR, a basic helix-loop-helix TF, is responsible for the regulation of genes involved in tapetal cell degradation, such as *OsCP1*, and mutations in *TDR* result in premature degradation of the tapetum and pollen sterility (Ji et al., 2013). EAT1 and TIP2 work synergistically with TDR to coordinate PCD timing and support pollen exine development, which is essential for male fertility in rice (Ko et al., 2014). TDR directly regulates genes involved in pollen wall development, including *OsC6* and *ADF*, by binding to their promoters (Li et al., 2015; Tariq et al., 2023). EAT1 works cooperatively with *TDR* to modulate the expression of *AP25* and *AP37*, regulators of tapetal PCD (Niu et al., 2013). TIP2 (bHLH142), which acts upstream of TDR and EAT1, coordinates tapetal degradation and pollen formation, and *tip2* mutants exhibiting delayed meiosis, defective tapetal PCD, and male Sterility (Fu et al., 2014; Ko et al., 2014). Additionally, the SLR1-MYB188 module integrates gibberellin signaling with PCD regulation, coordinating the expression of genes such as *CYP703A3*, *DPW*, *ABCG15*, and *PKS1*, all critical for pollen wall development (Jin et al., 2022). This regulatory interaction ensures that PCD occurs at the appropriate stage, allowing for proper deposition of sporopollenin and the formation of the pollen exine. Other transcription factors such as UDT1, OsMS1 and GAMYB are also involved in these regulatory pathways, with mutations leading to disruptions in tapetal development, resulting in pollen sterility (Jung et al., 2005; Liu et al., 2010; Yang et al., 2019).

In rice, nutrient synthesis and transport in the anther wall are vital for male fertility (Shi et al., 2011; Zhang et al., 2010). The most thoroughly researched directions collectively indicate that the formation of viable pollen depends on the synthesis and transport of lipid-based biomolecules, such as sporopollenin, Ubisch bodies, cuticular waxes, and cutin monomers. These compounds are essential for the proper formation of the pollen exine, which protects the pollen and ensures viability (Quilichini et al., 2015; Qin et al., 2013; Niu et al., 2013). Cytochrome P450 enzymes, including *CYP704B2* and *CYP703A3*, hydroxylate fatty acids essential for anther cuticle and pollen exine development (Li et al., 2010; Yang et al., 2014, 2018). Mutations in these enzymes disrupt pollen wall structure, leading to sterility. Similarly, *DPW* and *DPW2* regulate lipid accumulation and anther wax formation (Shi et al., 2011; Xu et al., 2017). Various ATP-binding cassette (ABC) transporters, such as ABCG15, ABCG26, and OsABCG3, are involved in the transport of lipid precursors for pollen exine and cuticle formation (Chang et al., 2018; Niu et al., 2013; Qin et al., 2013; Wu et al., 2014). Mutations in these transporters impair lipid transport and disrupt anther development, resulting in sterility (Zhang et al., 2010). Recent studies have further revealed that ROS homeostasis are jointly involved in the development of rice anther and panicles, namely, *DPS1* and *SUBSrP1* genes are considered to be crucial regulators of anther cuticle formation and ROS-mediated cell death in rice. *DPS1*, a cystathionine β-synthase (CBS) domain protein, maintains ROS homeostasis and cuticle integrity, with its loss-of-function mutants exhibiting increased ROS, early tapetal PCD, defective anther cuticles, and sterility (Zafar et al., 2020). Similarly, *SUBSrP1*, a subtilisin-like serine protease, is essential for anther cuticle biosynthesis in apical spikelets. Disruption of *SUBSrP1* leads to excessive ROS, cell death, impaired cuticle formation, and panicle degeneration (Ali et al., 2022). These studies collectively expand and demonstrate the importance of ROS homeostasis in male reproductive development and fruiting in rice.

Lipid metabolism genes such as *OsGPAT3* and *RSM2* are also integral to anther tapetal PCD regulation. *OsGPAT3* influences lipid metabolism within the anther, while *RSM2*, a GDSL lipase gene, impacts lipid metabolism in the anther wall (Men et al., 2017; Sun et al., 2018; Zhao et al., 2020). Other key genes, such as *WDA1*, *OsACOS12*, and *OsNP1*, are involved in sporopollenin biosynthesis and deposition, essential for pollen exine and anther cuticle formation. Mutations in these genes reduce the number of Ubisch bodies and oil bodies in the anther, leading to sterility (Chang et al., 2016a; Jung et al., 2006; Li et al., 2016; Liu et al., 2017). Additionally, *OsPKS1* and *OsPKS2*, homologs of Arabidopsis *PKSA*/*LAP6* and *PKSB*/*LAP5*, are critical for sporopollenin precursor biosynthesis. Mutations in *OsPKS2* result in sterility due to failed exine formation (Zhu et al., 2017; Zou et al., 2018).

In *Arabidopsis thaliana*, strictosidine synthase-like (*STR*-like) genes also play significant roles in plant metabolism, defense, and secondary metabolite pathways (Sohani et al., 2009). Glu-309, a key catalytic residue in strictosidine synthase, is crucial for catalysis, functioning as a proton donor. Some STR-like genes, including *AtSSL7* and *AtSSL14*, have diverged from their ancestral enzymatic functions and may serve regulatory roles in reproductive development (Kibble et al., 2009). Similarly, in rice, *OsSTRL2*, a homolog of the *Arabidopsis STR*-like genes, contributes to reproductive processes, particularly tapetal cell function and pollen development (Zou et al., 2017).

In this study, we identified a rice strictosidine synthase gene, *OsLAP3*, which is a new allele of the *OsSTRL2* gene and plays a critical role in anther cuticle formation and pollen exine patterning. Mutation of *OsLAP3* results in delayed tapetum degradation and defective pollen exine. Gene expression analyses revealed that *OsLAP3* is involved in lipid biosynthesis and transport, which are necessary for proper anther and pollen development. Although the *OsSTRL2* gene has been implicated in pollen development, its specific contributions to key biological processes such as lipid metabolism and tapetum degradation remain underexplored. In our study, we reported the *OsSTRL2/OsLAP3* gene through map-based cloning, and these findings not only provide a more comprehensive understanding of the molecular mechanisms of male sterility in rice but also offer a new potential gene resource for SPT system and practical application during in rice hybrid seed production.

## 2 Materials and methods

### 2.1 Plant materials and growth conditions

The *lap3* mutant was identified from a population of *indica* rice cv. Zhonghui8015 (Zh8015), generated through ^60^Coγ-ray radiation. For genetic analysis and mapping, the *lap3* mutant (*indica species*) was as the pollen acceptor in crosses with wild type (WT) Zh8015 and 02428 (*japonica species*). Heterozygous F_1_ plants were self-pollinated to produce BC_1_F_2_ and an F_2_ population, which were further utilized for segregation analysis. All rice plants were grown under natural conditions in paddy fields located at the China National Rice Research Institute, Hangzhou, Zhejiang Province and Lingshui, Hainan Province.

### 2.2 Phenotypic and cytological analysis

The phenotypic characteristics of the *lap3* mutant, including plant height, tiller number, spikelet morphology, and floral organs, were documented using a Nikon Coolpix E995 digital camera (Nikon, Tokyo, Japan) and a Carl Zeiss SteREO Lumar V12 stereo fluorescence stereomicroscope (Markku Saari, Jena, Germany). Pollen fertility was assessed by 1.0% I_2_/KI staining under a Leica DM2500 microscope. For histological analysis, anthers at various developmental stages were collected and processed for semi-thin sectioning, scanning electron microscopy (SEM), and transmission electron microscopy (TEM) (Sun et al., 2018, 2023).

### 2.3 Molecular cloning of *OsLAP3* and gene complementation analysis

For map-based cloning of *OsLAP3*, a mapping population of 1,196 male-sterile plants was selected from the F_2_ segregation population obtained by crossing *lap3* with 02428 (**Supplementary Table S2**). The modified cetyl trimethyl ammonium bromide (CTAB) method was used for DNA extraction (Allen et al., 2006). InDel markers were developed based on polymorphisms between the genome sequences of *japonica* Nipponpare and *indica* 9311 (http://www.gramene.org and http://blast.ncbi.nlm.nih.gov).

To verify the identity of the *OsLAP3* gene, a 5,745 bp genomic fragment was amplified, including a 2,643 bp promoter, a 2,238 bp coding region, and an 864 bp downstream region. The fragment was cloned into the binary vector pCAMBIA1300 (CAMBIA; hygromycin resistance) using the in-fusion HD cloning kit (Takara Bio USA Inc., Mountain View, CA, USA) and introduced into the *lap3* mutant through Agrobacterium-mediated transformation for complementation analysis. A CRISPR/Cas9 vector (pC1300-Ubi::Cas9-SK-5G), carrying rice Ubi promoter and a single-guide RNA (sgRNA), was constructed for targeting *OsLAP3* to develop its knock-out lines. A target sequence (5-GCTCGTGTCGGTGAAGAACA-3) with protospacer adjacent motif (PAM) sequence CCA was identified in the second exon of *OsLAP3*. Two oligos were designed using an online CRISPR-GE tool (http://skl.scau.edu.cn/). The possible off-target effects were prevented by BLAST search using target sequence in Gramene (www.gramene.org) database. Positive transgenic lines were identified by PCR, sequenced for confirmation, and analyzed for phenotypic changes. Primers used for vector construction and sequencing are listed in **Supplementary Table S3**.

### 2.4 RNA extraction and qPCR analysis

Total RNA was extracted from various rice tissues, including roots, stems, leaves, and anthers at different developmental stages, using the TRIzol reagent (Invitrogen, USA). cDNA synthesis was performed using a PrimeScript RT reagent kit (TaKaRa, Japan). Quantitative real-time PCR (qRT-PCR) was conducted using SYBR Premix Ex Taq II (TaKaRa, Japan) on a Roche LightCycler 480 system. Gene expression was normalized to *OsACTIN1* (*Os03g0234350*), and three biological replicates were included. Specific primers for each target gene are listed in **Supplementary Table S3**.

### 2.5 GUS histochemical staining

The promoter region of *OsLAP3* (2.2 kb upstream) was cloned into the pCAMBIA1305 vector to drive the expression of the GUS reporter gene. The pOsLAP3:GUS construct was introduced into Zh8015 via *Agrobacterium tumefaciens*-mediated transformation. GUS activity was detected in spikelets at various developmental stages by staining with a GUS staining kit. Stained anthers were decolorized using 70% ethanol and subjected to transverse sectioning for microscopic analysis. Primers for pOsLAP3:GUS construction are listed in **Supplementary Table S3**.

### 2.6 Phylogenetic analysis

The conserved domain of OsLAP3 protein was identified using the NCBI CD-search tool (https://www.ncbi.nlm.nih.gov/Structure/cdd/wrpsb.cgi). Homologous protein sequences were retrieved from the NCBI database, and multiple sequence alignments were conducted using ClustalW. A phylogenetic tree was constructed using the neighbor-joining method with MEGA10 software (bootstrap = 1000) to assess the evolutionary relationship between OsLAP3 and its homologs in other species.

### 2.7 Subcellular localization

To investigate subcellular localization, the full-length coding sequence of *OsLAP3*, excluding the stop codon, was cloned into the pCAMBIA1305 vector to generate an OsLAP3-GFP fusion protein. Rice mesophyll protoplasts were isolated from 2-week-old Zh8015 plants, and plasmids with either the recombinant or empty vector were transformed into the protoplasts. After incubation for 36 to 48 hours in the dark, GFP signals were examined using a Zeiss LSM700 confocal laser scanning microscope (Zeiss, Oberkochen, Germany). Co-localization analysis was performed using the ER marker PDI-mCherry. Primers for subcellular localization are listed in **Supplementary Table S3**.

### 2.8 TUNEL assay

To detect DNA fragmentation in the anthers in WT and lap3 mutant plants, the TUNEL (terminal deoxynucleotidyl transferase-mediated dUTP nick-end labeling) assay was performed according to a previously described method (Chen et al., 2018). Selected paraffin-embedded anther sections at different developmental stages were dewaxed in xylene and rehydrated in an ethanol series. The assay was conducted using an *in vitro* DeadEnd™ Fluorometric TUNEL System, using fluorescein (Promega, Wisconsin, USA), following the manufacturer’s instructions. The signals were observed and imaged using a fluorescence confocal scanner microscope (ZEISS LSM 700, Oberkochen, Germany) under consistent settings.

### 2.9 Analysis of wax and cutin components

Anthers from WT and *lap3* plants at the mature pollen stage, prior to heading, were collected for was cutin component analysis, with five biological replicates per sample. Extraction was performed as previously described in the Zhang Dabing Laboratory at Shanghai Jiaotong University (Li et al., 2010). The components were analyzed using gas chromatography–mass spectrometry (GC-MS), and the wax and cutin content between WT and mutant anthers was compared based on mass spectrometry results.

## 3 Results and analysis

### 3.1 The *lap3* mutant exhibits normal vegetative growth but complete male sterility

**Figure 1:**
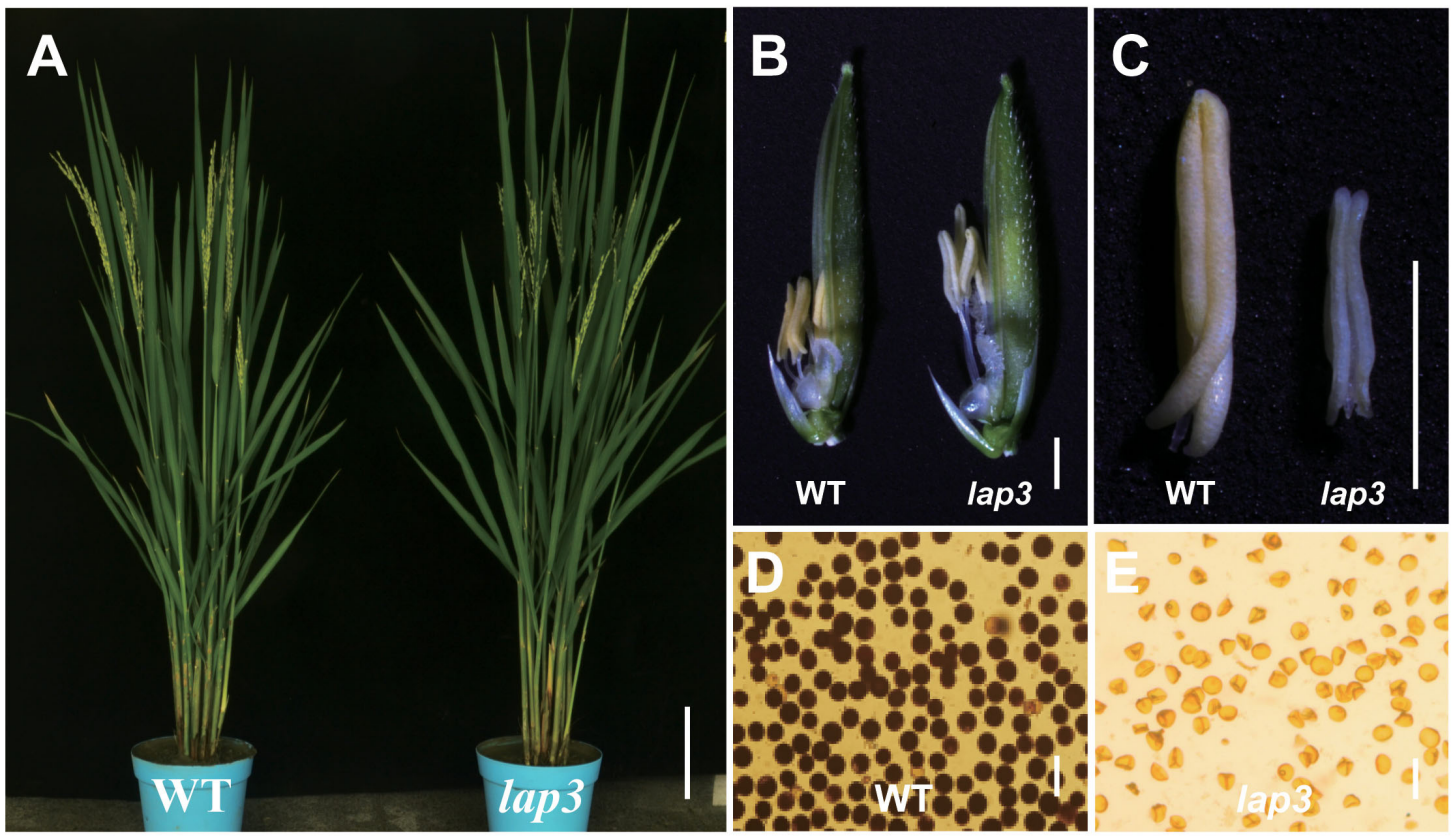
Phenotypes of the *lap3* mutant. **(A)** Wild type (WT) and the *lap3* mutant plants at anthesis stage. **(B)** Mature spikelets of WT Zh8015 and *lap3* mutant at anthesis; lemmas were removed for clarity. **(C)** Mature anther of WT and *lap3*. **(D, E)** Pollen fertility of WT and *lap3* by I_2_/KI staining. Bar = 15 cm **(A)**, 1.5 mm **(B)**, 500 µm **(C)**, and 10 µm **(D, E)**.

The *lap3* mutant displayed normal vegetative growth patterns, including comparable plant height, tiller number, and spikelet count to the WT Zh8015 (**Figure 1A**; **Supplementary Table S1**). Similarly, except for the morphology of the anthers, the number of flower organs in the mutant and the morphology of other flower organs, such as the lemma, palea and ovary, are basically the same as those in the wild type (**Figure 1B**). However, key differences were observed closer to the heading stage. In WT plants, the anthers were plump, golden, and shorter, while the stigmas were small, surrounded by filaments (**Figure 1B**). By contrast, the *lap3* mutant displayed significantly elongated filaments, larger stigmas, and smaller, shriveled, and white anthers (**Figure 1C**). Furthermore, I_2_/KI staining revealed that over 99% of WT pollen grains were fertile, as indicated by dark staining. In contrast, *lap3* mutants produced 100% abortive pollen, with no detectable staining (**Figures 1D, E**). These observations indicate that *lap3* mutants, while maintaining normal vegetative growth, exhibit complete male sterility due to defective anther and pollen development.

### 3.2 Abnormal tapetum and microspore development in *lap3*

**Figure 2:**
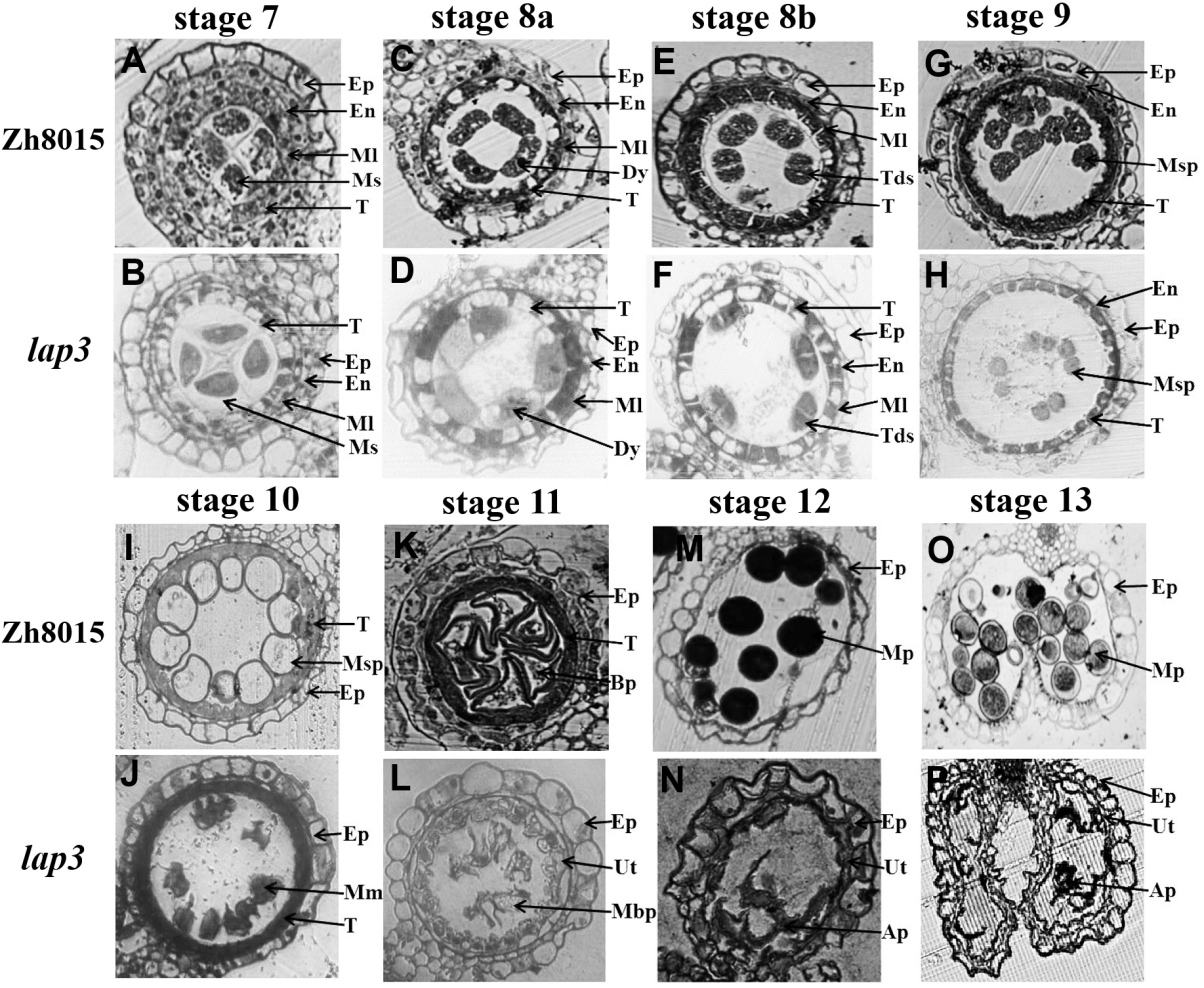
Transverse section analysis of anther development in WT and *lap3*. **(A, C, E, G, H, I, K, M, O)** and **(B, D, F, H, J, L, N, P)** are locules from anther sections of WT and *lap3* from stage 7 to stage 13, respectively. Ep, epidermis; En, endothecium; Ml, middle layer; T, tapetum; Ms, microsporocyte; Dy, dyad cell; Msp, microspore; BP, bicellular pollen; MP, mature pollen; Tds, tetrads; Mm, malformed microspore; Ap, abortive pollen; Ut, undegraded tapetum. Bars = 50 μm.

To further investigate the defects in the *lap3* anthers, semi-thin sections were analyzed at various developmental stages. At stage 7 (S7), no discernible differences were observed between WT (Zh8015) and *lap3* anthers; both contained well-formed microspore mother cells (**Figures 2A, B**; **Supplementary Figure S1**). However, by stage 8 (S8), the first signs of abnormal development appeared. While WT underwent normal meiosis to form tetrads (**Figures 2C, E**), *lap3* mutants showed irregular tetrad formation and failed to narrow and condense their tapetal cells (**Figures 2D, F**). At S9, WT tapetal cells underwent clear programmed cell death (PCD), leading to fragmentation and disintegration of the cell layer, whereas the *lap3* tapetal cells remained structurally similar to those observed at S8b (**Figures 2E, G, H**).

By S10, the differences were more pronounced. WT microspores became vacuolated, spherical, and were closely associated with the tapetal cells which had degraded (**Figure 2I**). In *lap3*, tapetal cells remained circular and microspores appeared irregularly shaped and scattered throughout the anther chamber (**Figure 2J**). By S11, the tapetum layer became more irregular and microspores had gradually degraded and underwent asymmetric mitosis, forming one generative cell and one vegetative cell in WT (**Figure 2K**). In contrast, the *lap3* tapetum had just begun to rapidly degrade, and the deformation and shrinkage of the microspores became more apparent (**Figure 2L**). By S12, WT microspores had formed bicellular pollen and contained two sperm nuclei and a vegetative nucleus (**Figure 2M**), the anther epidermis cracked normally, preparing for pollination (**Figure 2O**), but *lap3* anthers contained nearly hollow, collapsed pollen grains, with only remnants of microspores in the locule (**Figures 2N, P**).

### 3.3 Defective Ubisch body synthesis and abnormal pollen exine formation in *lap3*

**Figure 3:**
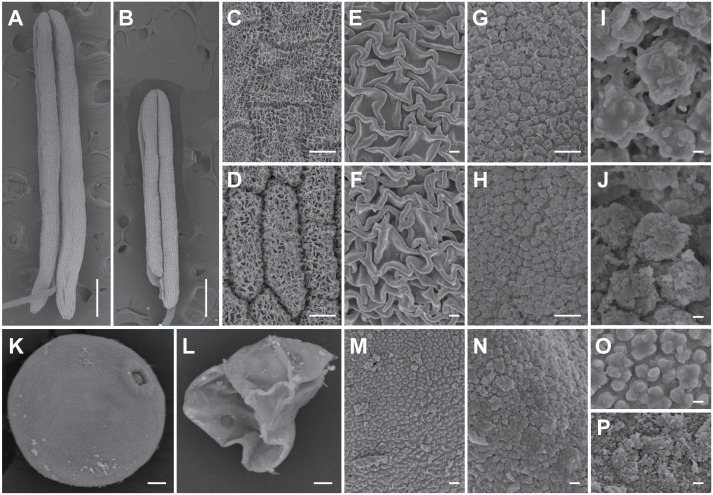
Scanning electron microscopy observation of anther and pollen grains in the WT and *lap3*. **(A, B)** Single anther of WT **(A)** and *lap3* **(B)** at stage 13. **(C-F)** Observation of anther cuticle on anther epidermis cells in WT **(C, E)** and *lap3* **(D, F)** at stage 13. **(G-J)** Observation on inner side of anther cells and Ubisch bodies in WT **(G, I)** and *lap3* **(H, J)** at stage 13. **(K, L)** Pollen grains of WT **(K)** and *lap3* **(L)** at stage 13. **(M-P)** Pollen exine and sporopollenin of WT **(M, O)** and *lap3* **(N, P)** at stage 13. Bars = 1 mm **(A, B)**; 20 µm **(C-H)**; 10 µm **(I-N)**; 200 nm **(O, P)**.

Scanning electron microscopy (SEM) was used to analyze the anther and pollen surface *lap3* mutant anthers were noticeably shorter (**Figures 3A, B**) and contained densely packed anther cuticle on anther epidermis cells than WT (**Figures 3C–F**). The inner walls of WT anthers showed a smooth, evenly distributed Ubisch bodies (**Figures 3G, I**). In contrast, the inner walls of *lap3* anthers were covered with underdeveloped, fluffy Ubisch bodies, indicating impaired sporopollenin deposition (**Figures 3H, J**). WT pollen grains were full and spherical, with smooth outer surfaces (**Figures 3K, M, O**), whereas *lap3* appeared shriveled and deformed, with an incomplete exine layer and abnormal sporopollenin deposition (**Figures 3L, N, P**).

### 3.4 Delayed tapetum degradation in *lap3*

**Figure 4:**
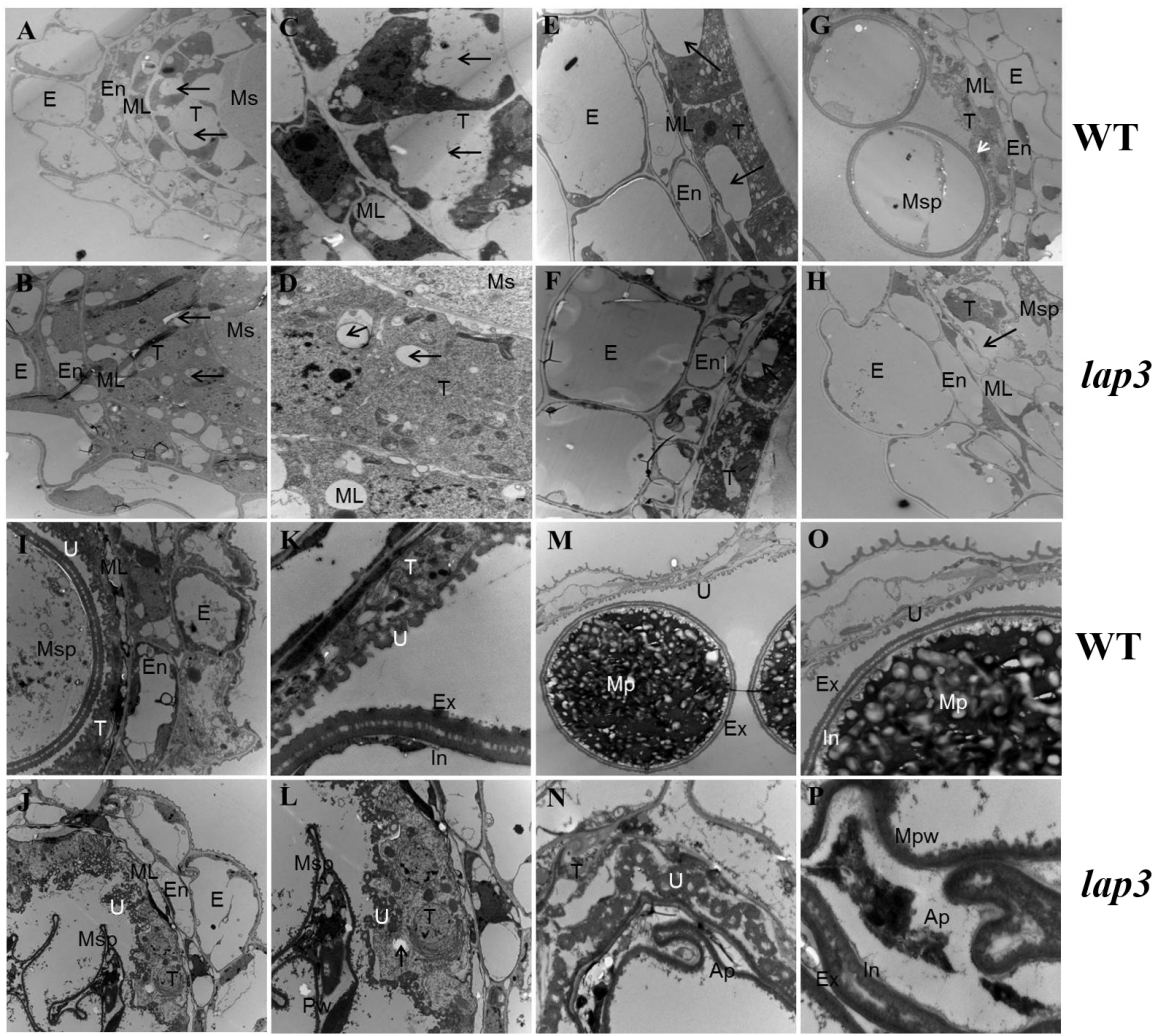
Transmission electron microscopy of anthers and pollen grains in WT and *lap3*. **(A, C, E, G, H, I, K, M O)** are from WT, and **(B, D, F, H, J, L, N, P)** are from *lap3*. **(A, B)** Anther wall layers and microsporocytes of WT **(A)** and *lap3* **(B)** at S7. **(C, D)** Higher magnification of WT **(C)** and *lap3* **(D)** tapetum at S7, showing nuclei and vacuoles. **(E, F)** Microsporocytes and tapetal cells with vacuole size differences (arrows) at S8b. **(G, H)** Anther wall structure of WT and *lap3* at S10. **(I, J)** Anther and tapetum structures at S11. **(K, L)** Higher magnification of microspores and tapetal cells at S11. **(M, N)** Tapetum cells, Ubisch body, and pollen exine at S12. **(O, P)** Higher magnification of M and N, respectively. Ap, abortive pollen; E, epidermis; En, endothecium; Ex, exine; In, intine; ML, middle layer; Mpw, malformed pollen wall; Ms, microsporocyte; Msp, microspore; T, tapetum; U, Ubisch body. Bars = 5 µm **(A, B, E-J, M, N)**; 0.5 µm **(C, D, K, L, O, P)**.

To pinpoint the timing and location of defects in anther development in the *lap3* mutant, semi-thin sectioning of anthers was performed at different developmental stages in both the WT and *lap3*, followed by TEM. At S7, the structural composition of the mutant anthers was similar to the WT, though the mutant’s tapetal cells were tightly packed with smaller vacuoles, while WT tapetal cells exhibited vacuoles occupying two-thirds of the cell. Nuclei appeared normal in both (**Figures 4A–D**). By S8b, WT anther epidermal cells had elongated shapes and orderly arrangement, whereas *lap3* mutant displayed disordered cuboidal epidermal cells. Although vacuoles in the mutant tapetal cells slightly increased in size, they remained significantly smaller than those in the WT (**Figures 4E, F**). At S10, the WT tapetum was thin and organized, supporting microspore vacuolization. In contrast, *lap3* tapetal cells exhibited large vacuoles and remained thicker, with irregularly shaped microspores distributed randomly in the locule (**Figures 4G, H**).

By S11, most WT tapetum had degraded, leaving regular Ubisch bodies in close contact with microspores, aiding nutrient transfer. The pollen wall separated into two layers following the first mitosis (**Figures 4I, K**). In *lap3*, the tapetum remained thick and unevenly distributed, with irregular Ubisch bodies. Microspores were irregular and shrunken, with no clear distinction between intine and exine layers of the pollen wall (**Figures 4J, L**). At the final stage of pollen maturation, WT pollen was filled with starch granules, while lap3 pollen became hollow, with thin walls and visible tapetal cells (**Figures 4M–P**). These findings suggest delayed tapetum degradation in lap3 led to nutrient deficiencies, disrupting pollen development, and causing sterility.

**Figure 5:**
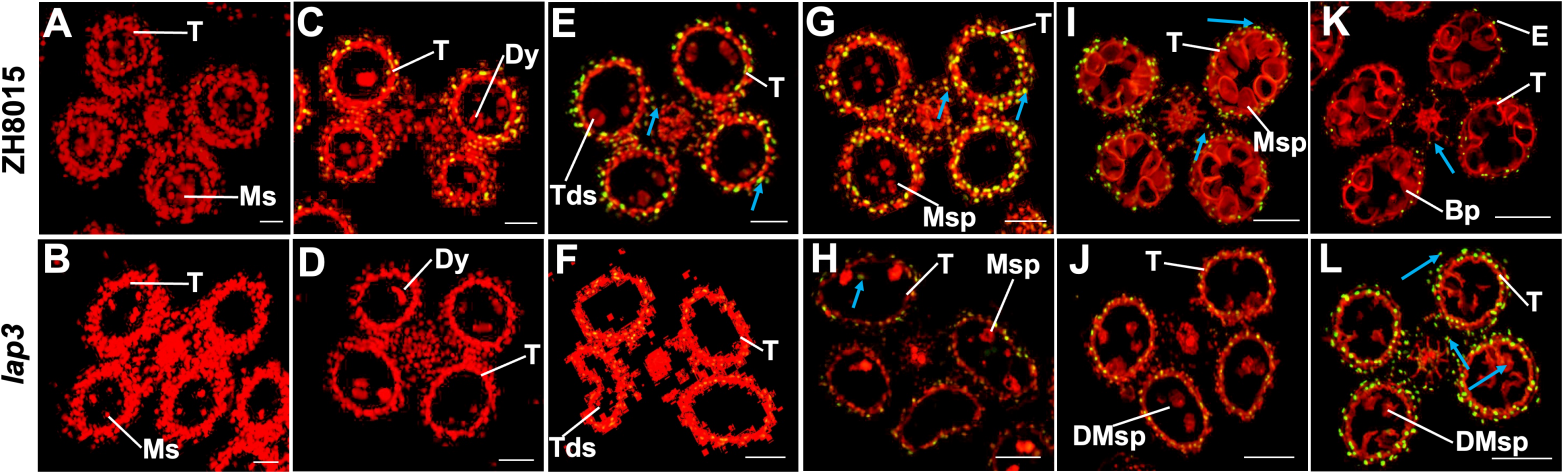
Detection of DNA fragmentation in WT(ZH8015) and *lap3* anthers by TUNEL assa**y. (A, B)** Anthers at stage 7. **(C, D)** Anthers at stage 8a. **(E, F)** Anthers at stage 8b. **(G, H)** Anthers at stage 9. **(I, J)** Anthers at stage 10. **(K, L) A**nthers at stage 12. Red signal indicates propidium iodide (PI) staining, and yellow and green fluorescence indicate TUNEL-positive signals. TUNEL-positive signals in tapetum cells are marked with white arrows, while those in outer cell layers (epidermis, endothecium, middle layer, and vascular bundle cells) are marked with blue arrows. BP, bicellular pollen; DMsp, degraded microspore; Dy, dyad cell; E, epidermis; Ms, microsporocyte; Msp, microspore; T, tapetum; Tds, tetrads. Bars = 50 µm.

To confirm delayed degradation, we performed TUNEL assays to detect DNA fragmentation, an indicator of PCD. At S7, no TUNEL signals were detected both in WT and *lap3* mutant (**Figures 5A, B**). In WT, strong TUNEL signals were detected in the tapetum at S8a and S8b, peaking at S9 and diminishing by S10, indicating near complete tapetum degradation, almost completely extinguished at S11 and S12 (**Figures 5C, E, G, I, K**). In *lap3*, no TUNEL signals were detected at S8a (**Figure 5D**) and S8b (**Figure 5F**), TUNEL signals appeared at S9 and persisted until S12, confirming delayed PCD and tapetal cell degradation (**Figures 5H, J, L**). This delay likely disrupted sporopollenin precursor release, contributing to abnormal pollen wall formation and male sterility in the mutant.

### 3.5 Genetic analysis and positional cloning of *OsLAP3*

Genetic analysis of F_1_, BC_1_F_2_, and F_2_ populations from a cross between the *lap3* and the WT showed that the male sterility phenotype in *lap3* is controlled by a recessive nuclear gene. Segregation analysis of BC_1_F_2_ and F_2_ populations revealed a 3:1 ratio of fertile to sterile plants, consistent with a recessive nuclear site of the mutation (**Supplementary Table S2**).

**Figure 6:**
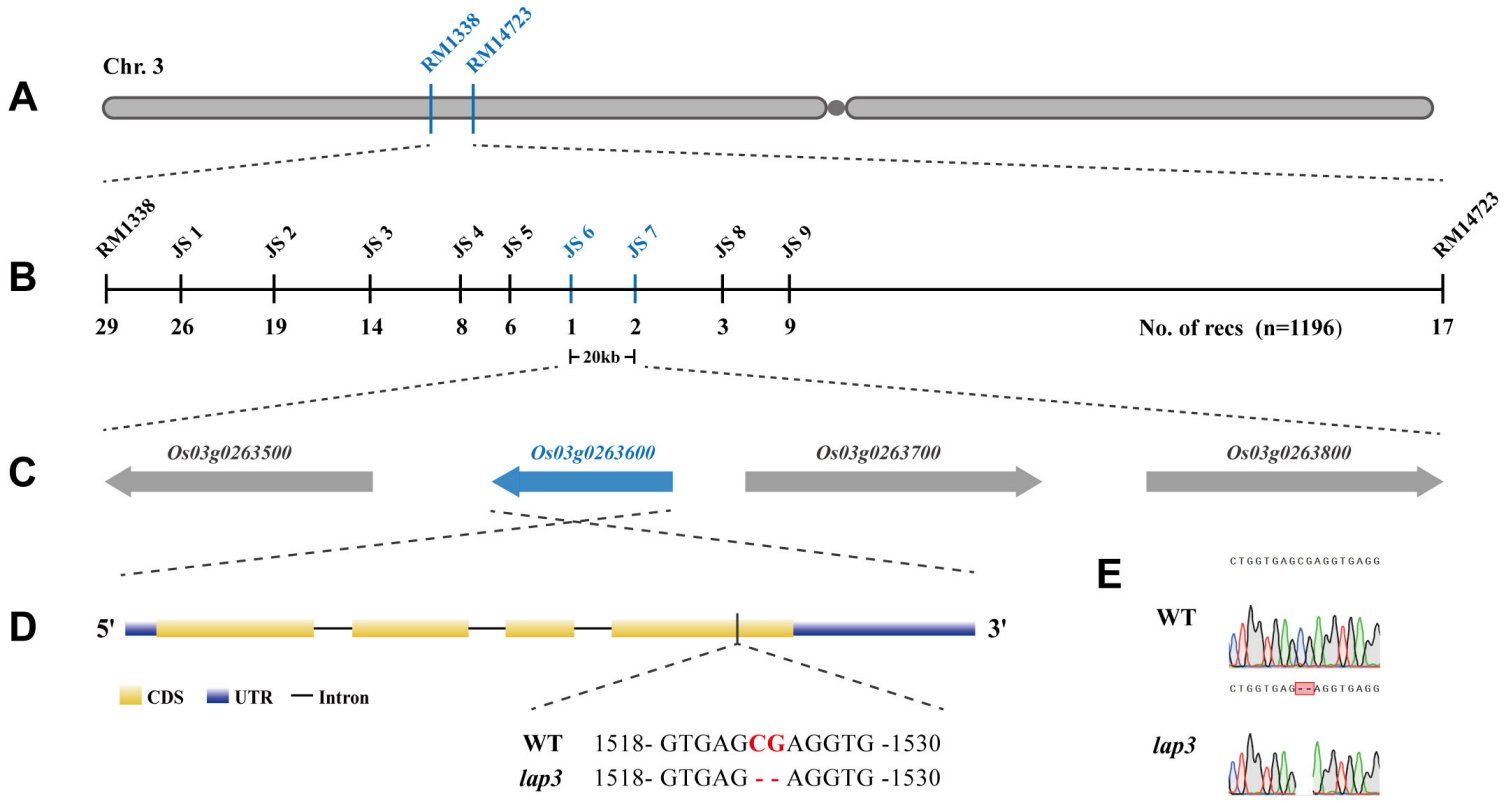
Fine mapping and molecular cloning of the *OsLAP3* gene. **(A)** Genetic linkage map of the *lap3* locus between *RM1338* and *RM14723* on the short arm of chromosome 3. **(B)** Fine mapping of *lap3* to a 20 kb genomic region between JS6 and JS7. **(C)** Four ORFs in the target region; the red one was the target gene. **(D, E)** Sequence analysis of *OsLAP3*. Red capitals in Panel D and red frame in E represent the differential nucleotide base between WT and *lap3 mutant*.

For positional cloning of the *OsLAP3* gene, 1,196 sterile plants from the F_2_ population were used for map-based cloning. Initial mapping with InDel markers localized *lap3* to a region on the short arm of chromosome 3, between markers RM1338 and RM14723 (**Figure 6A**). Fine mapping further narrowed the region to a 20 kb interval between markers JS6 and JS7 (**Figures 6B, C**). Sequence analysis identified a two-nucleotide deletion in the *Os03g0263600* gene (**Figures 6D, E**), which resulted in a frameshift and premature termination of the protein (**Supplementary Figure S3**). This confirmed *Os03g0263600* as the candidate gene for *OsLAP3*. The gene has a full length of 2,083 bp, consisting of 4 exons and 3 introns (**Supplementary Figure S3A**). The coding region is 1,284 bp in length and encodes a 427 amino acid protein containing: a six-bladed beta-propeller TolB-like domain and a strictosidine synthase domain (**Supplementary Figure S3B**). The deletion in *lap3* caused a substitution of position 390, changing serine (Ser) to arginine (Arg), resulting in premature termination of translation (**Supplementary Figure S3C**).

### 3.6 Functional verification of *OsLAP3*

**Figure 7:**
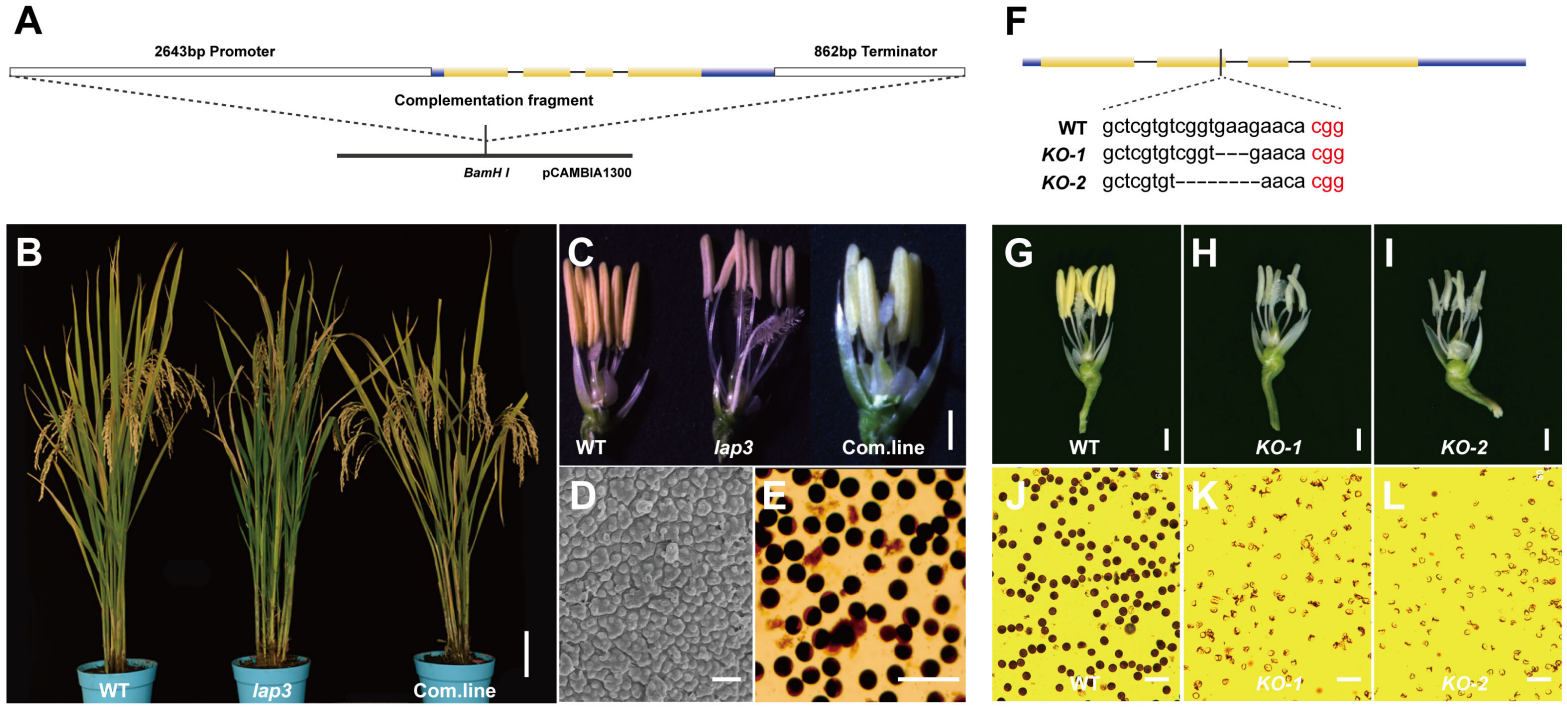
Function complementation of *OsLAP3*. **(A)** DNA fragment used for complementation. **(B) S**eed setting rate of spikelet in WT, *lap3*, and complementation plants. **(C)** Flowers and anther at stage 13 of WT, *lap3*, and complementation plant. **(D)** Pollen exine surface in complementation plants by SEM. **(E)** OsLAP3-complemented mutants stained with 1% I_2_-KI solution showing mature pollen grains. **(F)** OsLAP3 knockout target and mutation type of KO plants. **(G-I)** Flowers and anthers of WT and *lap3*-Cri plants at stage 13. **(J-L)** WT and *lap3*-Cri plants stained with 1% I_2_-KI solution showing mature pollen grains. Bars = 1 mm **(C)**; 200 nm **(D, E)**.

To confirm that *OsLAP3* is responsible for the male sterility in *lap3*, a 5.75 kb genomic fragment containing the *OsLAP3* coding region was introduced into *lap3* mutants via *Agrobacterium*-mediated transformation (**Figure 7A**). Hygromycin resistance screening identified positive transgenic plants, which were subsequently grown in the field. Sampling and observation at the heading and flowering stages revealed that the complemented transgenic lines exhibited restored fertility, with yellow viable pollen, and similar seed set rates to WT plants (**Figures 7B–E**). SEM further confirmed the restoration of the WT pollen exine structure in the transgenic lines, verifying *OsLAP3* function (**Figures 7D, E**).

Additionally, the CRISPR-Cas9 knockout experiment of *OsLAP3* generated *lap3*-Cri plants with 3 bp and 5 bp deletions (**Figure 7F**). Both KO-1 and KO-2 lines exhibited milk-yellow anthers (**Figures 7H, I**) and sterile pollen (**Figures 7K, L**) compared to wild-type yellow anthers (**Figure 7G**) and dark I2/KI stained pollen (**Figure 7J**). These results further demonstrated that *OsLAP3*, encoding strictosidine synthase domain protein, is responsible for the male sterile observed in the *lap3* mutant.

### 3.7 Phylogenetic analysis of *OsLAP3*

**Figure 8:**
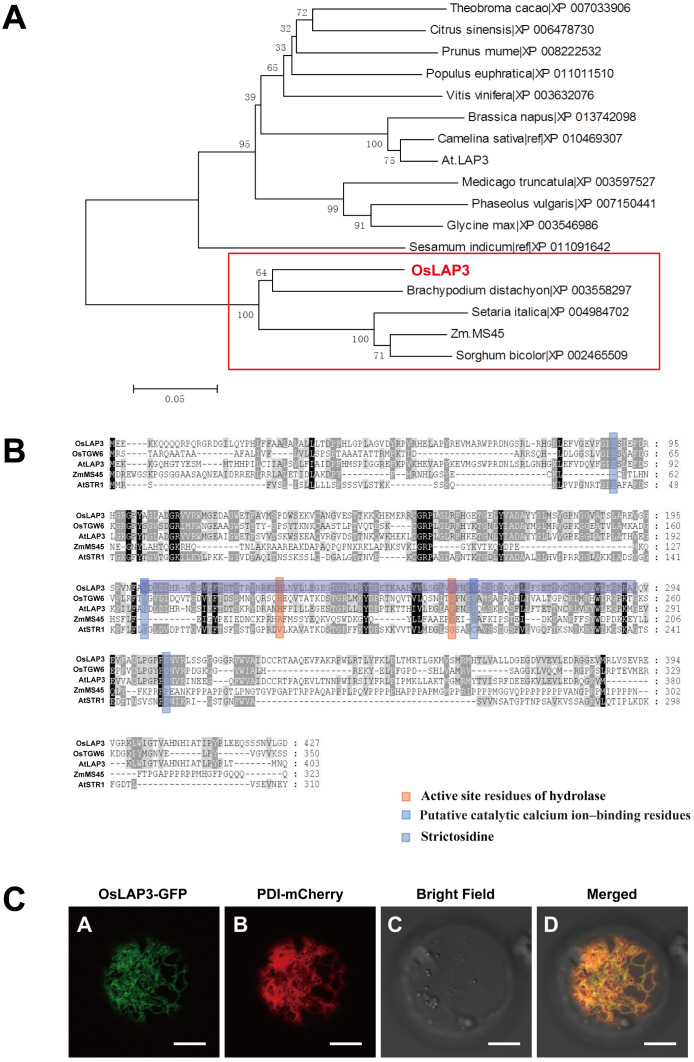
Functional analysis of OsLAP3 protein. **(A)** Homology analysis and protein sequence alignment of OsLAP3. **(B)** Evolutionary analysis of OsLAP3 and homologous proteins. **(C)** Subcellular localization of OsLAP3-GFP fusion protein and ER marker PDI-mCherry in rice protoplasts.

Phylogenetic analysis of *OsLAP3* and its homologs in other plant species revealed that *OsLAP3* belongs to the strictosidine synthase gene family, situated within a distinct evolutionary branch of the Poaceae family among monocots. *OsLAP3* is most closely related to the strictosidine synthase gene in *Brachypodium distachyon*, indicating a high degree of homology (**Figures 8A, B**). With the poaceae family, the only reported strictosidine synthase gene is *ZmMS45*, which primarily regulates the development of the anther tapetum and pollen exine (Zhang et al., 2014). This function is similar to the phenotype observed in the *lap3* mutation. Additionally, its homologous gene *AtLAP3* has been reported to have similar functions (Dobritsa et al., 2009), suggesting that *OsLAP3* plays a conserved role in regulating male reproductive development across different plant species.

To determine the subcellular localization of *OsLAP3*, a GFP-tagged *OsLAP3* construct was transiently expressed in rice protoplasts. Confocal laser scanning microscopy revealed that *OsLAP3* predominantly localizes to the endoplasmic reticulum (**Figure 8C**; **Supplementary Figure S4**).

### 3.8 Expression analysis of *OsLAP3*

**Figure 9:**
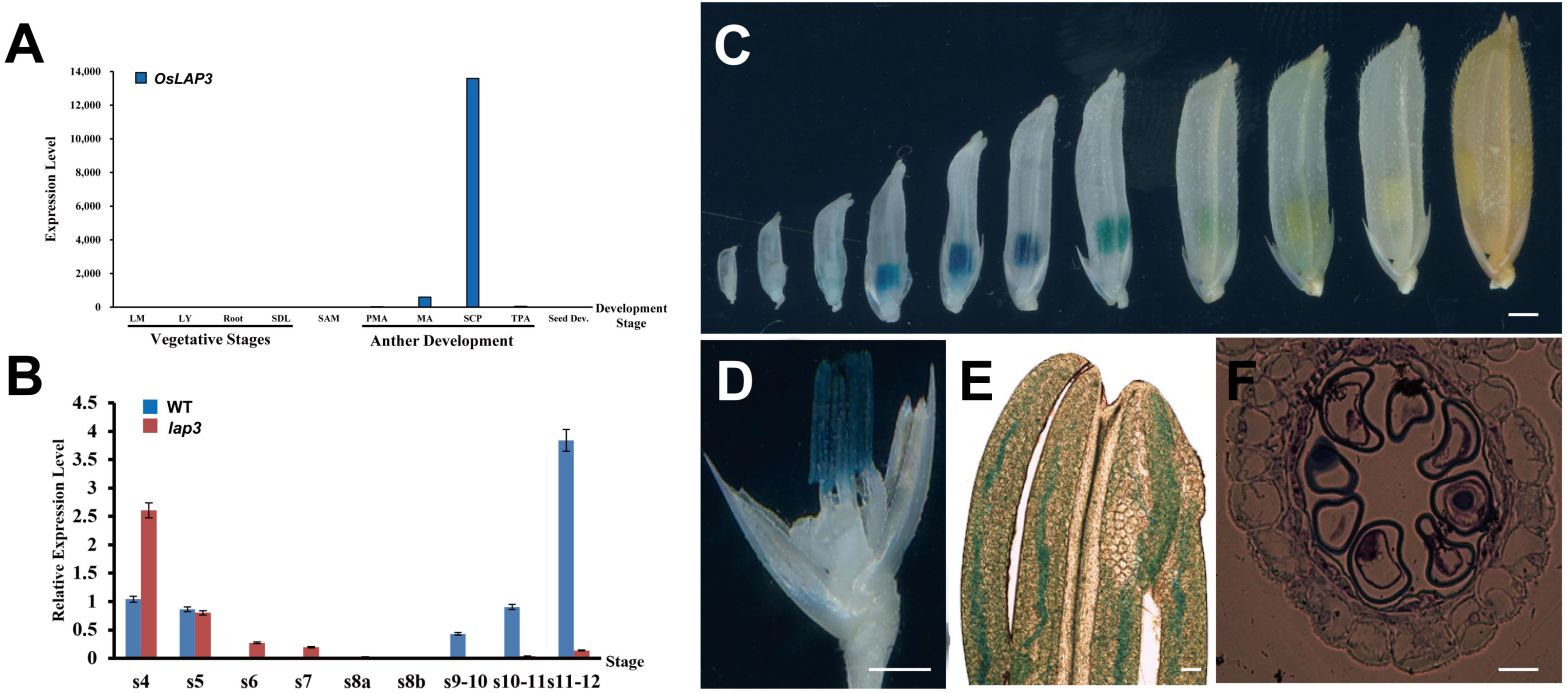
Expression analysis of *OsLAP3.* **(A)** Expression of *OsLAP3* in different tissues. **(B)** Expression level in WT and *lap3* mutant anthers at various development stages. **(C)** GUS expression patterns (blue staining) in the heterozygous spikelets of *OsLAP3*-promoter-GUS transgenic lines at various stages. **(D)** GUS staining of an 8 mm spikelet (palea and lemma removed, marked by a red arrow). **(E)** Phase-contrast microscope analysis of anther from **(D, F)** Transverse section of anther from **(D)** Bars = 0.5 mm **(C-E)**; 20 µm **(F)**.

Given that the *lap3* mutant specifically affects rice anther development and pollen fertility, with minimal impact on vegetative growth and floral organ development, quantitative real-time PCR (qRT-PCR), promoter-GUS fusion protein transgenic analysis, and *in situ* hybridization were employed to examine *OsLAP3* expression during anther development. Initially, qRT-PCR analyzed *OsLAP3* expression in various tissues developmental stages of the anther. *OsLAP3* expression level was almost undetectable in low in roots, stems, flag leaves, and leaf sheaths (**Figure 9A**). However, expression was observed during meiosis and mitosis in anther development, with peak expression during mitosis. Further analysis of *OsLAP3* expression in the anthers at different development stages in WT and mutants revealed that during the early meiosis phase (from S5 to S7), *lap3* expression in the mutant was slightly higher than in the WT. After microsporocyte formation and the onset of mitosis, *OsLAP3* expression in the WT significantly increased, while in the mutant, it markedly declined. This trend continued until stage S12 (**Figure 9B**), consistent with previous observations from semi-thin sections and electron microscopy.

GUS staining analysis of transgenic plants carrying an *OsLAP3*-promoter-GUS construct demonstrated that *OsLAP3* was barely expressed in florets and anthers measuring 1–5 mm. However, strong expression was observed exclusively in the anthers of 6–9 mm florets, peaking at 7–8 mm florets (**Figure 9C**). Semi-thin section analysis indicated that this stage corresponded to the late S10 of anther development (**Figures 9D–F**). Staining was predominantly concentrated in the anther wall, with weaker staining in the microspores. As floret length exceeded 9 mm, *OsLAP3* expression began to decrease, and in nearly mature florets, expression became undetectable. These findings suggest that *OsLAP3* plays a crucial role in regulating anther wall development and pollen maturation during the mitotic phase.

### 3.9 *lap3* mutant anthers show reduced wax and cutin content

**Figure 10:**
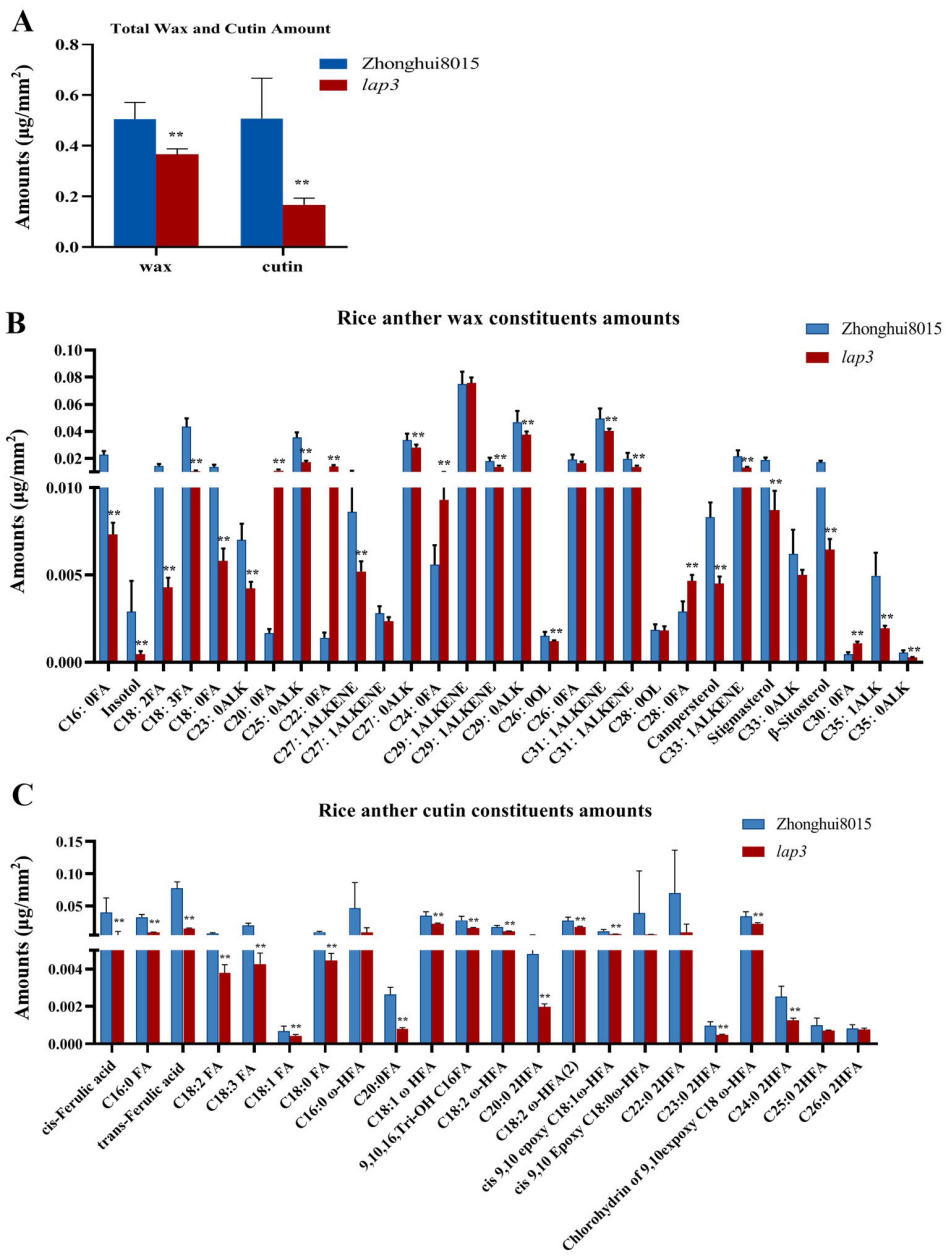
*OsLAP3* mutation reduces wax and cutin content in anthers. **(A)** Total wax and cutin contents in WT and *lap3* anthers. **(B)** Wax component contents. **(C)** Cutin component contents. Data are mean ± SD of five biological replicates (**p < 0.01). C16:0 FA, hexadecanoic acid; C18:0 FA, octadecanoic acid; C18:1 FA, oleic acid; C18:2 FA, linoleic acid; C18:3 FA, linolenic acid; C20:0 FA, eicosanoic acid; C22:0 2HFA, 2-hydroxy-docosanoic acid; C24:0 FA, tetracosanoic acid; C26:0 FA, hexacosanoic acid; C28:0 FA, octacosanoic acid; C30:0 FA, triacontanoic acid,; ALK, alkane; C24:0 2HFA, 2-hydroxy-tetracosanoic acid; C25:0 2HFA, 2-hydroxy-pentacosanoic acid; C18:0 ω-HFA, cis-9,10-epoxy 18-hydroxy-stearic acid; C18:2 ω-HFA, 18-hydroxy-linoleic acid; C18:1 ω-HFA, cis-9,10-epoxy-18-hydroxy-oleic acid; C26:0 2HFA, 2-hydroxy-hexacosanoic acid; C16:0 ω-HFA, 16-hydroxyhexadecanoic acid.

The synthesis and accumulation of lipid polymers are essential for anther development and pollen function in rice. Cuticular wax and cutin, which are major components of the anther epidermis, contribute to maintaining structural integrity. Disruptions in these pathways often lead to male sterility due to defects in anther epidermis and pollen wall formation. To investigate the impact of the *lap3* mutation on lipid metabolism in rice anthers, wax and cutin compositions were analyzed in both WT and *lap3* anther (**Supplementary Figure S2**). The results showed notable changes in wax and cutin profiles in the *lap3* mutant, highlighting the broad effects of this mutation on lipid biosynthesis. Total wax content in *lap3* anthers was significantly reduced by 42.65%, with a level of 0.5391 µg/mm² compared to 0.9398 µg/mm² in WT anthers (**Figure 10A**). Among wax components, C18:0 FA (stearic acid) showed a significant reduction, while C24:0 FA (lignoceric acid) displayed an increase, potentially indicating a compensation mechanism in long-chain fatty acid metabolism. Additionally, plant sterols such as β-sitosterol and stigmasterol were significantly reduced in *lap3*, potentially affecting membrane stability (**Figure 10B**). Cutin content also showed a 22.95% reduction in lap3, from 0.3328µg/mm² in WT to 0.2564 µg/mm² in the mutant. Key cutin monomers, such as C18:1 ω-HFA and 9, 10, 16 Tri-OH C16FA, were significantly reduced in *lap3* (**Figure 10C**), suggesting that the mutation affects specific steps in cutin biosynthesis.

These data indicate that the *lap3* mutation disrupts multiple lipid biosynthesis pathways, affecting both wax and cutin accumulation. The altered lipid profiles in *lap3* suggest compromised anther epidermis integrity, which may contribute to its reproductive deficiencies. These findings highlight the importance of *OsLAP3* in maintaining the balance of these components for normal anther and pollen development.

### 3.10 Expression analysis of *OsLAP3*

**Figure 11:**
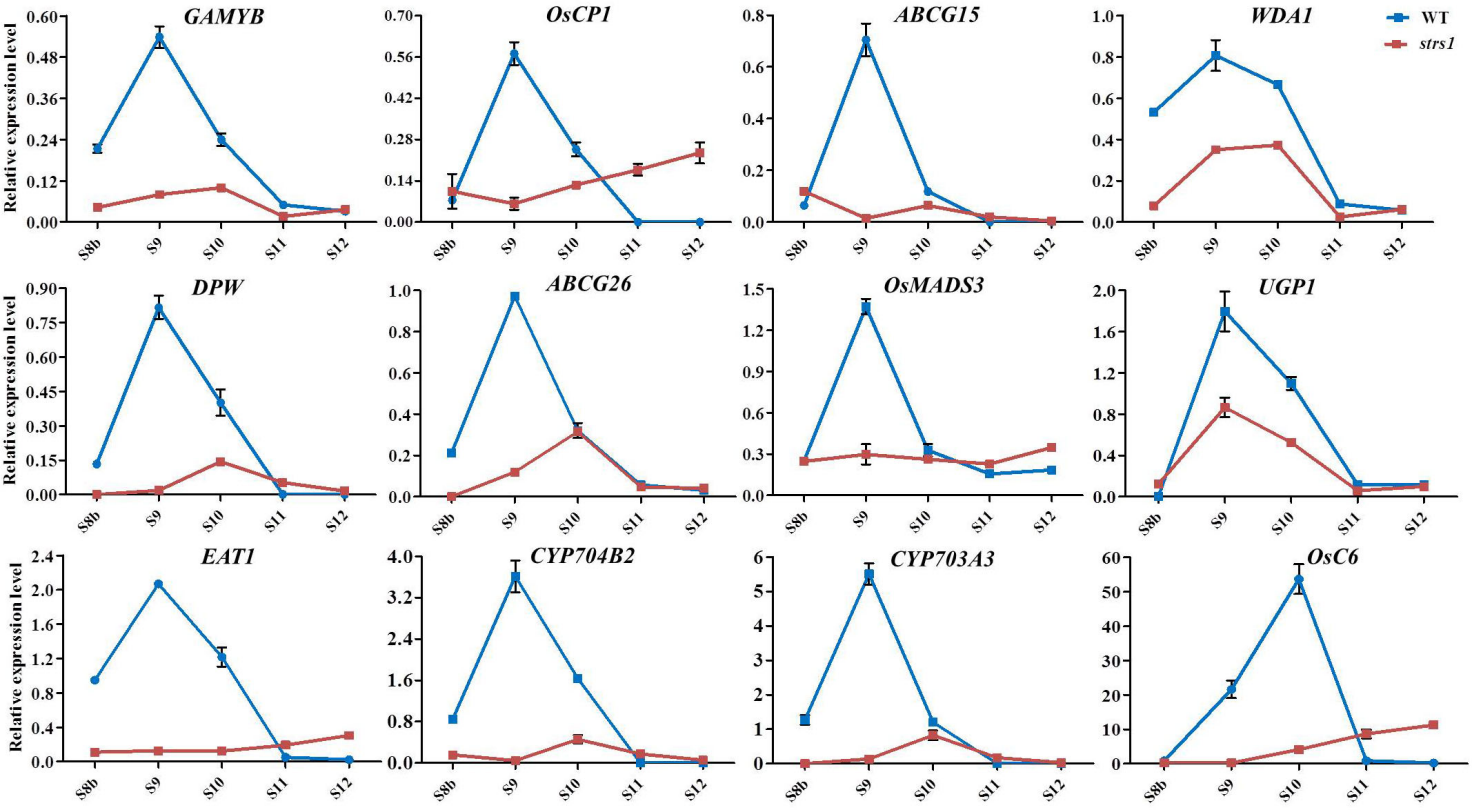
Expression profile of the genes involved in anther development in WT and *lap3*. *OsActin1* was used as an internal control. Error bars show the SD (n = 3). S8b. stage 8b; S9. stage 9; S10. stage 10; S11. stage 11; S12. stage 12; WT. wild type.

Gene expression patterns related to anther and pollen development were compared between both WT and *lap3* mutant plants at different stages of anther development (S8b to S12) using qRT-PCR (**Figure 11**). The expression levels of 12 key genes involved in these processes, including *GAMYB*, *OsCP1*, *ABCG15*, *WDA1*, *DPW*, *ABCG26*, *OsMADS3*, *UGP1*, *EAT1*, *CYP704B2*, *CYP703A3*, and *OsC6*, were analyzed. In WT anthers, sharp increases in gene expression, peaking at S9 and S10. For example, *GAMYB*, *OsCP1*, and *WDA1* exhibited the highest expression at S9, while *OsC6* peaked at S10. This peak expression coincides with critical stages of tapetum development and pollen wall formation, indicating the involvement of these genes in these processes. In contrast, the *lap3* mutant exhibited significantly lower expression of these genes throughout all stages, suggesting a disruption in transcriptional regulation.

Notably, genes involved in lipid biosynthesis for pollen exine formation, such as *CYP704B2* and *CYP703A3*, and those in tapetum PCD, such as *EAT1* and *OsMADS3*, showed much lower expression in lap3 compared to WT plants. Some genes, such as *OsC6*, exhibited slight increases in expression during later stages (S11 and S12) in *lap3*, possibly indicating a compensatory response to the developmental abnormalities. However, this late upregulation was insufficient to rescue the mutant phenotype, highlighting the importance of timely gene expression for proper anther and pollen development.

These expression patterns suggest that *OsLAP3* plays a crucial role in regulating tapetum degradation and the biosynthesis and transport of lipids during function of strictosidine synthase genes in male reproductive development across plant species.

## 4 Discussion

In this study, *OsLAP3* was characterized and identified as a gene essential for anther cuticle formation and pollen exine patterning in rice. The *lap3* mutant exhibited normal vegetative growth but displayed complete male sterility, characterized by shrunken, sterile anthers and defective pollen. Through genetic, cytological, and molecular analyses, it was demonstrated that *OsLAP3* plays a critical role in regulating lipid biosynthesis, tapetum degradation, and sporopollenin deposition during pollen development. These findings underscore the importance of *OsLAP3* in maintaining tapetum integrity and enabling proper pollen maturation.

### 4.1 Functional comparative analysis

Distinct roles of *OsLAP3* and *OsSTRL2* in anther development were highlighted in this research, both of which belong to the strictosidine synthase-like family. *OsLAP3* was found to significantly impact lipid metabolism and the timely degradation of tapetal cells, processes crucial for anther development and pollen viability. Mutations in *OsLAP3* were shown to disrupt fatty acid biosynthesis pathways and delay PCD in tapetal cells, as confirmed by lipid profiling and TUNEL assays. This disruption led to aberrant lipid changes that impaired pollen exine formation. It was further established that key regulatory genes like *CYP704B2*, *CYP703A3*, and *OsC6*, which are integral to lipid biosynthesis and sporopollenin formation, are directly influenced by *OsLAP3*, establishing its critical role in pollen fertility. In contrast, *OsSTRL2* was associated with broader anther dysplasia, without a specific link to lipid pathways, reflecting its atypical functional profile (Yang et al., 2018; Zou et al., 2017).

### 4.2 *OsLAP3* is essential for lipid biosynthesis and pollen exine formation

*OsLAP3* was found to be involved not only in fatty acid synthesis and transport but also in the PCD of tapetal cells. Future research will focus on identifying the upstream regulatory factors of *OsLAP3*. As an enzyme gene, *OsLAP3* likely acts downstream, while its upstream regulators are thought to be transcription factors.

*OsLAP3* was shown to coordinate multiple signaling pathways to regulate rice male reproductive development by modulating a series of genes. In the lap3 mutant, qPCR results and phenotypic analysis revealed that the expression of several lipid metabolism-related genes, such as *CYP704B2*, *CYP703A3*, and *OsC6*, was significantly downregulated. *CYP704B2* and *CYP703A3*, which catalyze the hydroxylation of C16/C18 fatty acids and the modify lauric acid, respectively, play critical roles in sporopollenin biosynthesis (Li et al., 2010; Yang et al., 2014). Mutations in *OsC6*, a lipid transport protein, leads to abnormal Ubisch bodies and defective pollen wall structures, indicating that *OsLAP3* regulates these genes to ensure proper anther development. The regulatory role of *OsLAP3* was shown to be selective within lipid biosynthesis pathways. While genes associated with sporopollenin and essential wax and cutin components exhibited significant changes in expression and accumulation, other lipid components, such as C24:0 fatty acid, remained stable or even increased, likely due to compensatory adjustments in long-chain fatty acid metabolism. This selective regulation ensures that *OsLAP3* primarily affects key components crucial for anther epidermis and pollen wall integrity, while non-essential components are maintained through redundant metabolic pathways or compensatory mechanisms, preserving basic metabolic functions.

Tapetum PCD is closely related to pollen wall formation, with evidence suggesting that *OsLAP3* coordinates these processes by regulating PCD-related genes such as *OsCP1* and *OsC6*. TUNEL assays indicated a significant delay in PCD in the *lap3* mutant, suggesting a role for *OsLAP3* in regulating the PCD process and, thus, impacting anther development. *OsLAP3* may interact with PCD-related genes, such as *TDR* (*Tapetum Degeneration Retardation*), which controls the expression of genes including *OsCP1* and *OsC6*, ensuring proper tapetal degradation and pollen wall formation (Li et al., 2015). Additionally, *SLR1* (*Slender Rice 1*) was found to interact with *MYB188* to regulate several downstream genes involved in sporopollenin biosynthesis, including *CYP703A3*, *DPW*, and *ABCG15*, which are critical to pollen wall formation (Jin et al., 2022). We hypothesize that *OsLAP3* is regulated by multiple transcription factors, including bHLHs and the SLR1-MYB188 module, which will be the focus of future research. Through these regulatory mechanisms, *OsLAP3* ensures timely tapetal PCD and the synthesis and transport of lipids during anther development, providing structural and nutritional support for normal pollen wall formation.

### 4.3 Functional conservation of strictosidine synthase domain proteins in reproductive development

Strictosidine synthase domain-containing proteins are known for their roles in secondary metabolite biosynthesis in plants, particularly in alkaloid production (Stöckigt et al., 2008). However, their function in reproductive development has been less explored. In this study, *OsLAP3* was shown to share homology with strictosidine synthase proteins from various plant species, including *Brachypodium distachyon* and *Zea mays*, both involved in reproductive development. *MS45* in *Zea* mays, for example, has been implicated in regulating tapetal development and pollen wall formation (Wang et al., 2022). Additionally, two strictosidine synthase genes, *AtLAP3* and *OsSTRL2*, have been reported to be involved in male reproductive development (Zou et al., 2017). In our study, *OsLAP3* is involved in lipid biosynthesis and pollen exine formation, showing the conserved function of strictosidine synthase-like genes in plant reproductive development. The conserved function of *OsLAP3* in rice, similar to these genes, suggests its role as a core regulator in male sterility systems.

Comparing the roles of *OsLAP3* and *OsSTRL2* (Zou et al., 2017) in pollen wall formation and tapetal cell regulation revealed similarities and differences that enhance the understanding of their functions in rice reproductive biology. Both genes are key regulators of anther development and play essential roles in pollen exine formation. High conservation was observed with *AtLAP3* and *ZmMS45*, both involved in sporopollenin biosynthesis, a crucial component of the pollen wall. *OsLAP3* loss-of-function resulted in defective pollen development and male sterility, similar to *OsSTRL2.* However, *OsLAP3* demonstrated a more refined regulatory mechanism compared to *OsSTRL2*, particularly in its coordination of tapetum PCD. This network is believed to operate more effectively in *OsLAP3* due to its ability to interface with multiple pathways, enhancing its regulatory accuracy in pollen exine and cuticle formation. The expression patterns of *OsLAP3*, predominantly in anthers and particularly in tapetal cells and microspores during pollen development, further supported its significant role in integrating multiple developmental cues.

### 4.4 *OsLAP3* acts as a potential target for GMS line breeding and rice hybrid seed production

The development of male sterility systems has been critical for hybrid seed production in rice, particularly in overcoming the limitations of conventional CMS (cytoplasmic male sterility) and P/TGMS (photo/thermo-sensitive genic male sterility) systems (Li et al., 2007). Despite the widespread use of CMS, low utilization efficiency has been reported due to the dependence on fertility restorer genes, while P/TGMS lines have been shown to be vulnerable to environmental fluctuations, leading to inconsistent seed purity. These limitations have prompted the exploration of more efficient sterility systems. To address these challenges, innovative technologies such as Seed Production Technology (SPT) have been developed to improve seed production efficiency and stability (Chang et al., 2016b). *OsLAP3*, given its functional similarity to *ZmMS45*, has emerged as a promising candidate for next-generation rice male sterility systems. *ZmMS45* has been successfully used in maize hybrid seed production as a core component of the smart sterility system (Wu et al., 2016). Similarly, *OsLAP3* was identified as playing a pivotal role in regulating tapetum PCD and pollen wall formation, making it an ideal target for developing hybrid rice production systems that leverage its regulatory functions. Collaborations with Boleyn Genetics have enabled the integration of *OsLAP3* into SPT technology, aimed at developing stable, non-GMO male sterile lines for hybrid seed production. The potential of *OsLAP3* in improving rice productivity through GMS line breeding and its application in SPT systems is significant, with future developments expected to further enhance hybrid rice breeding technologies.

## Data Availability

The datasets presented in this study can be found in online repositories. The names of the repository/repositories and accession number(s) can be found in the article/**Supplementary Material**.
