# Supplementary material for: OsLAP3/OsSTRL2, encoding a rice strictosidine synthase, is required for anther cuticle formation and pollen exine patterning in rice
Source: Front Plant Sci. 2025 Jan 20;15:1508828. doi: 10.3389/fpls.2024.1508828 (PMC11789761; doi:10.3389/fpls.2024.1508828)
Supplement: Supplementary file 1 [file DataSheet1.docx]

**Supplementary information:**

**
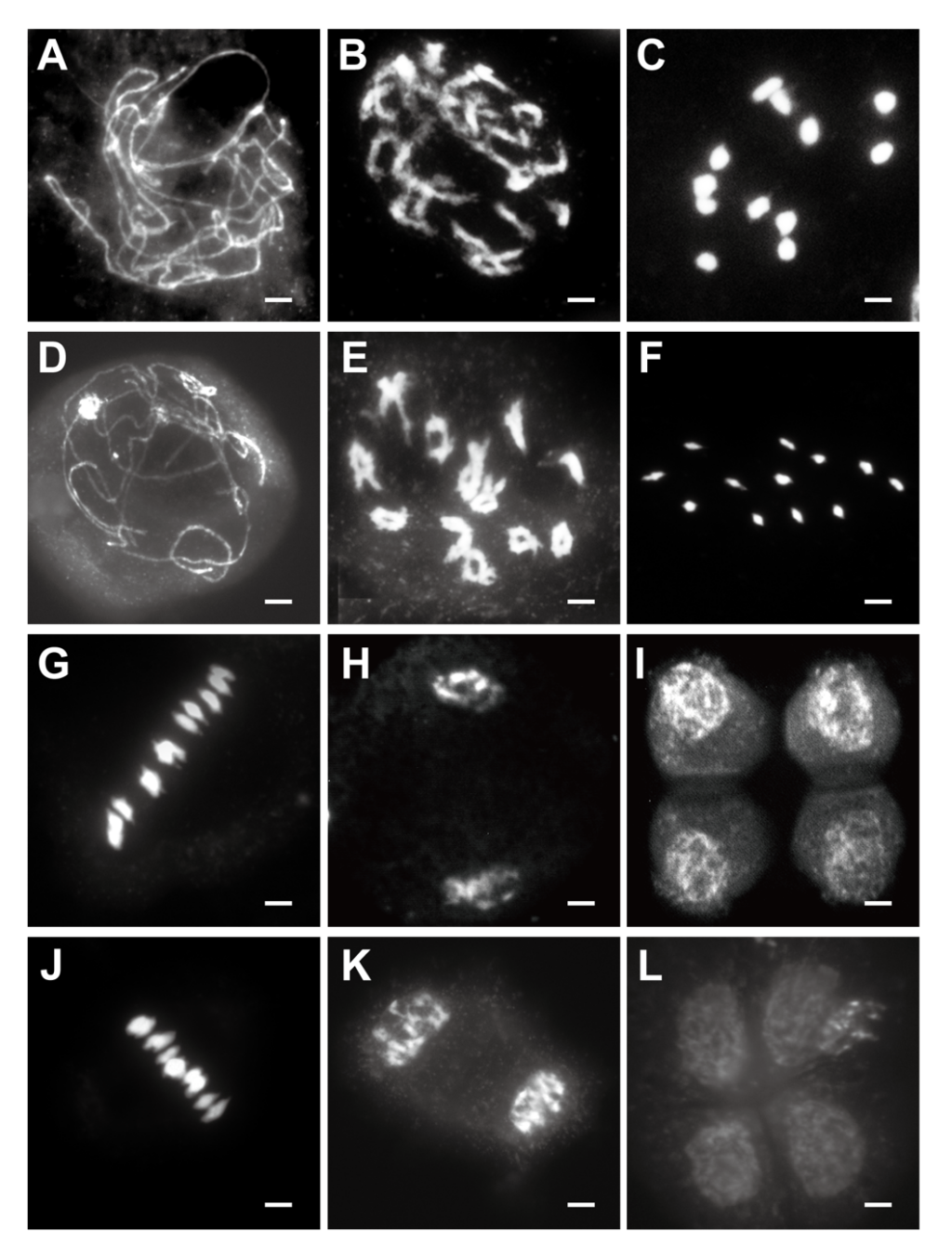
**

**Figure S1.** Chromosome behavior of male meiocytes of wild type ZH8015 and *lap3* at different stages by DAPI staining.

(A) and (D) ZH8015 and *lap3* meiocytes at pachytene;(B) and (E) ZH8015 and *lap3* meiocytes at diplotene;(C) and (F) ZH8015 and *lap3* meiocytes at diakinesis;(G) and (J) ZH8015 and *lap3* meiocytes at metaphase I ;(H) and (K) ZH8015 and *lap3* meiocytes at dyad stage;(I) and (L) ZH8015 and *lap3* meiocytes at tetrad stage .Bars=5 μm.

**
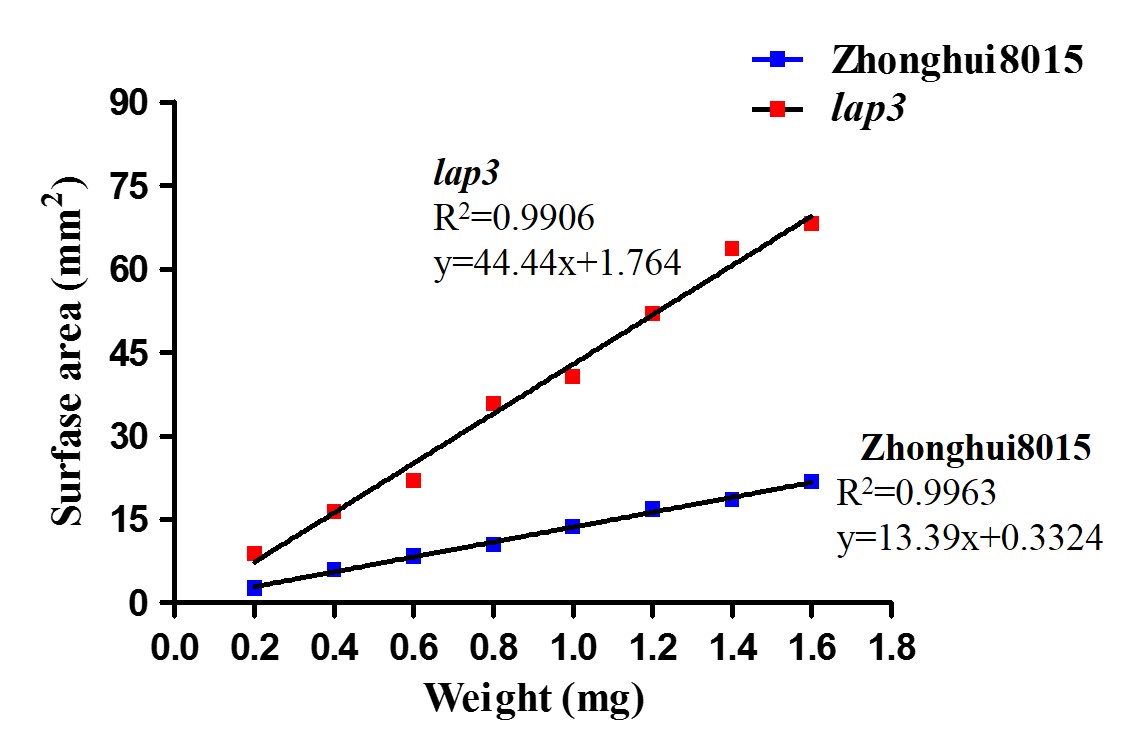
**

**Figure S2.** The ratio of weight/surface area of the anthers in the wild type Zhonghui8015 and the *lap3* mutant.

**
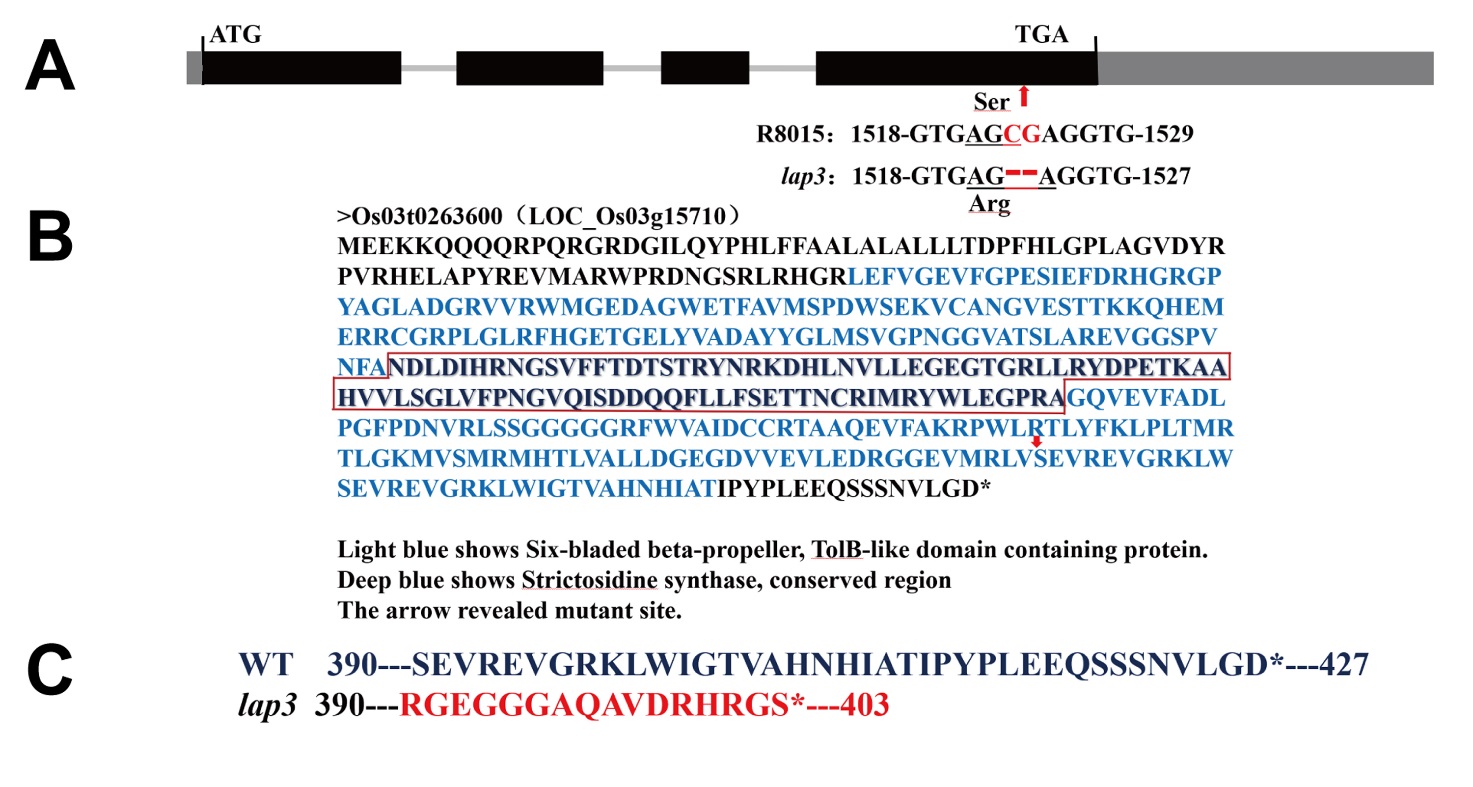
**

**Figure S3.** Sequence analysis of the *lap3* mutation site.


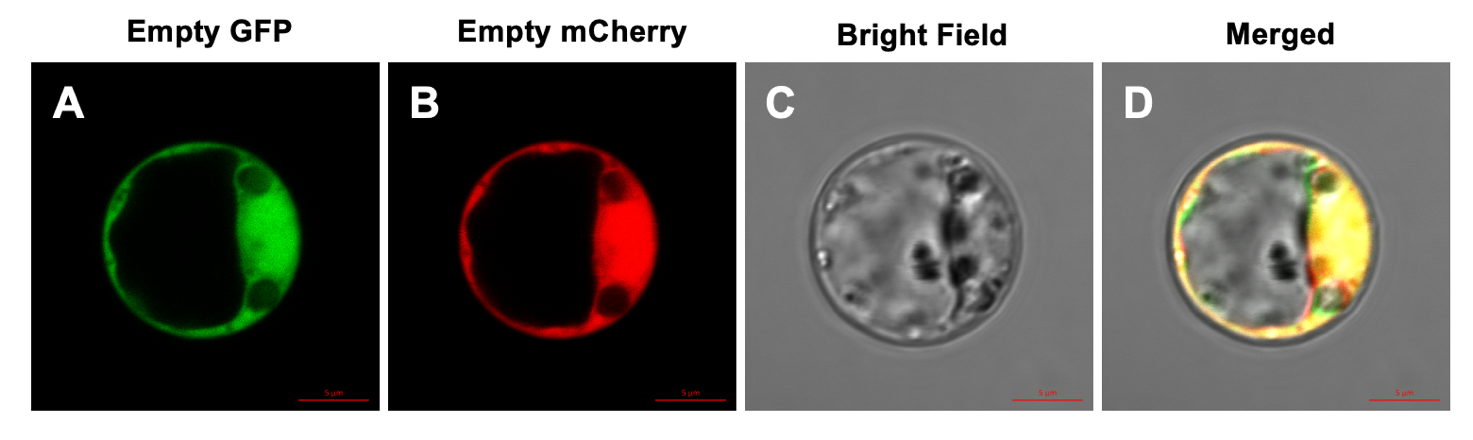


**Figure S4.** Negative control for subcellular localization of LAP3 protein

**Table S1. Comparison of agronomic traits between ZH8015 and *lap3***

| Agronomic Traits | ZH8015 | *lap3* | *P* |
| --- | --- | --- | --- |
| Plant height(cm) | 108.0±2.7 | 104.5±1.9 | 0.0002 |
| Tiller number | 9.0±0.7 | 8.8±0.4 | 0.6075 |
| Days to heading | 98.2±2.7 | 101.2±2.8 | 0.0005 |
| Spikelet Count | 125.7±6.4 | 126.8±5.2 | 0.4039 |
| Seed setting rate(%) | 84.3±1.6 | 0 | <0.0001 |

| **Table S2. Segregation analysis of the *lap3* allele** | | | | | |
| --- | --- | --- | --- | --- | --- |
| **Combination** | **Seed-setting**  **rate of F_1_** | **F_2_** | | **χ^2^(3:1)** | **χ^2^_0.05_** |
|  |  | **No. of wild type plants** | **No. of mutant plants** |  |  |
| *lap3*/Zh8015 | 88.21 | 385 | 123 | 0.17 | 3.84 |
| *lap3*/02428 | 85.78 | 3904 | 1256 | 1.19 |  |

**Table S3.** Primer sequences used in this study

| Primer | Forward primer (5′-3′) | Reverse primer (5′-3′) | Purpose |
| --- | --- | --- | --- |
| RD0304 | GGCGTCACTGCTCGTA | GCCTGAAGCGTCCACA | Linkage analysis |
| RM1338 | TGCAAGTTGGACTTCAAAGAGG | TGGATTCCTTCTTCCTTTCTCTCC |  |
| RM14723 | GCAAAGTCCTTTGGACAGGTAGC | CGTCCCAGATCAAAGTACACTCTTCC |  |
| RM7 | TTCGCCATGAAGTCTCTCG | CCTCCCATCATTTCGTTGTT |  |
| JS-1 | GGCCTCTGATACTACTCGATCTCG | TGCTCGACCAAATGCTCACG | Fine mapping |
| JS-2 | GAGGCAATAGCAGCGGACG | GCCAACGGCAGAGGTCTTC |  |
| JS-3 | CTGTGCAACTAGTCCGTTAAGAC | CTACTTAAATGTATAGATTAGGG |  |
| JS-4 | ATACAAGCAATGTGGCGTGGA | CAAACACGGGTCACATCCACT |  |
| JS-5 | TCTAACCGGTGTATTTCGTGT | CCGTCAGAAGTTTTGACAATG |  |
| JS-6 | TCAAGGCAATGCACTTACTAAC | AATCGTATTTTCTCAGGAACGA |  |
| JS-7 | CGGCCGGATTACGGACATT | TCTGCCGTTTCCTCACCAAT |  |
| JS-8 | AAATAAGACTGACGGTTAAACG | AAGTTCTAGAGGGAATGAGCAT |  |
| JS-9 | TTCTTGGTCTGGATTGGAGTG | TTTGTCATCTGTTCCATAGGC |  |
| LAP3-1 | TAATGCGGTACTGGCTGGAA | TAATGCGGTACTGGCTGGAA | Sequencing |
| LAP3-COM-*BamH*Ⅰ | CGGTACCCGGGGATCCCCTCCCTCGATTTGTACACGG | CGACTCTAGAGGATCCTTTCCTCGAAAAGGTCGGTAACT | Complementation vector construction |
| GP-Cas-*Aar*Ⅰ | AGATGATCCGTGGCAgctcgtgtcggtgaagaacacggGTTTTAGAGCTATGC | | CRISPR/Cas9 vector construction and Sequencing |
| Cas-Seq | CTCTCGTAGCAGTGTACAAT | GTCATATCTGAGCAGCCTC |  |
| OsLAP3-ISH-Anti Sense | CGAGAGAAGTCGGCGGGAG | TAATACGACTCACTATAGGGTCCAGCCAGTACCGCATTATC | In situ hybridization |
| OsLAP3-ISH-Sense | TAATACGACTCACTATAGGGCGAGAGAAGTCGGCGGGAG | TCCAGCCAGTACCGCATTATC |  |
| OsLAP3-GUS | GGACTCTTGACCATGGGAATTTGCACGCAGAGGAAAGA | TACCGAGCTCGAATTCCGGGTAGGCCAACGAATTCAC | GUS vector construction  (*Nco*Ⅰ/*EcoR*Ⅰ) |
| OsLAP3-GFP | GCCCAGATCAACTAGTCAATTCCGTCTTTCCTCTGC | TGCTCACCATGGATCCATCACCAAGCACGTTGCTGCTAC | GFP vector construction  (*Spe*Ⅰ/ *BamH*Ⅰ) |
| *OsLAP3* | CGACACGAGCACGAGATA | ACGCCATTCGGGAAGACCA | qPCR |
